# Shear Bond Strength of Biointeractive Restorative Materials to NeoMTA Plus and Biodentine

**DOI:** 10.3390/polym17223070

**Published:** 2025-11-20

**Authors:** Zübeyde Uçar Gündoğar, Gül Keskin, Merve Yaman Küçükersen

**Affiliations:** 1 Department of Pediatric Dentistry, Faculty of Dentistry, Gaziantep University, 27310 Gaziantep, Türkiye; 2 Department of Pediatric Dentistry, Faculty of Dentistry, Alanya Alaaddin Keykubat University, 07400 Alanya, Türkiye; 3 Inci Dis Dental Clinic, 34416 Istanbul, Türkiye

**Keywords:** pediatric dentistry, bioceramics, biointeractive materials, shear bond strength, adhesion

## Abstract

Background: The bonding compatibility between calcium silicate-based bioceramic cements and restorative materials is critical for long-term success in pediatric dentistry. This study compared the shear bond strength (SBS) of contemporary biointeractive restorative materials to two widely used bioceramics, NeoMTA Plus (NM) and Biodentine (BD). Methods: Eighty acrylic resin blocks with standardized cavities were filled with either NM or BD (*n* = 40 each) and subdivided into four restorative groups: nanohybrid composite (Filtek Ultimate), giomer (Beautifil II), bioactive restorative (Activa BioActive Restorative), and high-viscosity glass ionomer cement (Fuji IX GP Extra) (*n* = 10 each). All restorations followed a standardized etch-and-bond protocol. SBS was measured using a universal testing machine, and failure modes were assessed under a stereomicroscope. Data were analyzed using one-way ANOVA and Tukey’s HSD (*p* < 0.05). Results: BD exhibited significantly higher SBS values than NM (*p* < 0.001). In the BD group, Filtek Ultimate and Beautifil II achieved the highest and statistically comparable SBS, outperforming Activa BioActive Restorative and Fuji IX GP Extra (*p* < 0.05). In the NM group, no significant differences were found among materials. Adhesive failures predominated in NM (85%), while BD showed more cohesive failures (50%). Conclusions: Biodentine demonstrated superior bonding stability to restorative materials, with composite resin and giomer performing best. Giomer’s bioactivity and ion release make it a viable alternative to composite resin in suitable clinical contexts.

## 1. Introduction

Restorative dentistry has evolved beyond traditional approaches that focus primarily on replacing lost tooth structure, placing greater emphasis on biologically based and minimally invasive practices [1]. Particularly in pediatric patients, preserving pulp vitality, supporting tissue regeneration, and establishing long-lasting, functional tooth-restoration interfaces are critical for the success of treatment [2]. The high caries risk, limited cooperation, and biological sensitivity of developing dental tissues in pediatric patients present additional clinical challenges in selecting restorative materials and techniques [3]. In this context, advances in biomaterial technology and a deeper understanding of the biological processes of dental tissues play a crucial role in the adoption of new treatment approaches, particularly in pediatric dental practice. Today, the success of restorative treatments is no longer evaluated solely by addressing mechanical deficiencies but also by maintaining biological integrity and achieving long-lasting restorations that closely resemble natural tissue [4,5].

Calcium silicate-based bioceramic cements have emerged as biocompatible materials that enhance the success of vital pulp therapies, particularly in managing pulp injuries and deep carious lesions commonly encountered in pediatric and endodontic dentistry [6]. These cements are bioactive and hydrophilic materials that set via hydration reactions, release calcium and hydroxyl ions to promote mineralization, and exhibit antibacterial and sealing properties due to their high alkalinity and dentin-like dimensional behavior [7]. Mineral trioxide aggregate (MTA), one of the well-established materials in this group, has gained a prominent place in clinical practice due to its high biocompatibility and its ability to support tissue healing; however, it presents certain limitations, such as a prolonged setting time and potential for tooth discoloration [8,9]. Neo MTA Plus (Avalon Biomed Inc., Houston, TX, USA) is a powder/gel-based calcium silicate cement specifically formulated to overcome the limitations of traditional MTA, such as tooth discoloration and prolonged setting time. Composed primarily of tricalcium and dicalcium silicate without bismuth oxide—instead using tantalum oxide as a radiopacifier—Neo MTA Plus offers enhanced color stability, superior handling, and reliable bioactivity [10,11,12]. Another clinically relevant bioactive cement is Biodentine (Septodont, Saint-Maur-des-Fossés, France), a high-purity calcium silicate-based material developed with patented Active Biosilicate Technology. Composed primarily of high-purity tricalcium silicate and zirconium oxide, it exhibits superior biological and physical properties compared with conventional bioceramics. Its alkaline pH promotes hard tissue formation and pulp vitality while its formulation ensures fast setting, improved handling, and biocompatibility without cytotoxic effects [13,14]. The long-term success of vital pulp therapy depends not only on the performance of the bioceramic material but also on the properties of the overlying restoration and the integrity of the interface between the two materials. A strong, durable bond is essential to prevent microleakage and bacterial infiltration, which can cause restoration failure and loss of pulp vitality [2,15].

Recent advances in restorative materials have provided clinicians with several restorative options for various clinical situations. Composite resins, as direct restorative materials, are widely used for anterior and posterior restorations owing to their excellent clinical performance [16,17]. Although composite resin technology continues to advance, there is a growing interest in bioactive restorative materials that not only provide mechanical stability but also interact with dental tissues by releasing beneficial ions, such as calcium, phosphate, and fluoride, to promote remineralization and exhibit antibacterial effects [18]. Bio-interactive materials trigger a biological response at the interface, forming a bond between tissue and material. By releasing ions, they deliver essential minerals, bind to collagen, promote apatite crystallization, and help protect tissues from degradation. These materials also maintain a favorable pH for mineral formation and inhibit bacterial growth [7]. Among these materials, high-viscosity glass ionomer cements (HV-GIC) stand out with their improved mechanical and biological properties compared with classical glass ionomers. By reducing particle size and increasing the powder–liquid ratio, HV-GICs exhibit improved reaction rates and mechanical durability while their ionic exchange layer formed with dentine enables sustained fluoride release and promotes dentine remineralization, thereby reducing the risk of caries [19]. Building on the developments in glass ionomer technology, giomer (Beautifil II, Shofu Inc., Kyoto, Japan) has emerged as an innovative restorative material that incorporates prereacted glass ionomer (PRG) fillers into a resin-based matrix. These bioactive fillers are capable of both releasing and recharging beneficial ions such as fluoride, strontium, and borate that contribute to tooth remineralization, reduce acid production by cariogenic bacteria, and help inhibit plaque accumulation. Moreover, giomer supports bio-interactivity by promoting apatite formation at the resin–dentin interface, thus improving the durability of restorations through long-term tissue–material interaction [20,21]. More recently, a flowable, bioactive, resin-based restorative material (Activa BioActive-Restorative, Pulpdent Corporation, Watertown, MA, USA) has been developed, combining the benefits of composite resins and glass ionomer cement. According to the manufacturer, Activa BioActive-Restorative is the first dental product to feature an ionic resin matrix, a shock-absorbing resin component, and bioactive fillers designed to promote remineralization and hydroxyapatite formation in surrounding tissues [21,22]. The bioactivity of the material is attributed to its ability to respond to pH fluctuations by releasing and reabsorbing calcium, phosphate, and fluoride ions [23].

These innovations in restorative materials highlight the changing landscape of restorations and the importance of exploring their bonding effectiveness. The proper bond between bioceramic cement and the overlying restorative material is a crucial clinical factor for the long-term success of the restoration. Numerous studies have investigated the adhesive properties and bonding efficacy of various restorative materials to bioceramics [24,25,26,27]. However, there is limited conclusive information about these, possibly due to the wide variety of restorative and base materials, bonding procedures, and clinical conditions. In pediatric dental practice, there is an ongoing need for scientific evidence regarding which restorative materials form stronger and more durable bonds with bioceramics. Therefore, the current study aimed to evaluate and compare the shear bond strength (SBS) and failure modes of two different bioceramic cements (Neo MTA Plus and Biodentine) bonded to four restorative materials: nanohybrid composite, giomer, bioactive restorative material, and high-viscosity glass ionomer cement. The null hypothesis was that there would be no significant differences in SBS between the two bioceramic materials, regardless of the type of restorative material used. The findings are expected to inform the selection of materials and development of treatment protocols in biologically oriented, minimally invasive restorative dentistry, particularly for pediatric patients. To our knowledge, studies evaluating SBS using the specific combinations of bioceramic cements and restorative materials selected in this study are limited.

## 2. Materials and Methods

### 2.1. Study Design and Power Analysis

This in vitro experimental study was conducted to evaluate the SBS of four different restorative materials to two bioceramic cements. Sample size was determined a priori with G*Power (version 3.1.9.7, Universität Düsseldorf, Düsseldorf, Germany). For the primary analysis (one-way ANOVA comparing four restorative subgroups within each bioceramic; α = 0.05), a medium-to-large effect (Cohen’s f = 0.46) and 90% power indicated approximately 10 specimens per subgroup [28]. For the overall comparison of NeoMTA Plus versus Biodentine (independent-samples *t*-test; α = 0.05, power = 0.80), a moderate-to-large difference (Cohen’s d ≈ 0.64) supported approximately 40 specimens per group. A post-hoc sensitivity analysis was planned to report the minimum detectable effects with the achieved sample sizes (ANOVA: Cohen’s f; between-material contrast: Cohen’s d) at 80% power. Ethics committee approval was not required for this in vitro study; the work adhered to internationally accepted standards for the ethical conduct of research.

### 2.2. Specimen Preparation

In this study, four different restorative materials and two bioceramic cements were evaluated. The composition and application procedures for each material are detailed in Table 1. Products were within expiry and sourced from official distributors; lot numbers were not recorded during experimentation. All procedures were performed at standard room temperature (22 ± 2 °C) and ambient humidity (50 ± 10%).

A total of 80 acrylic resin blocks, each with a diameter of 5 mm and a central hole measuring 2 mm in height, were prepared. To ensure a smooth and horizontal surface, the blocks were meticulously trimmed using a trimmer. The acrylic blocks were then randomly divided into two groups based on the bioceramic used: Neo MTA Plus (*n* = 40) [NM] and Biodentine (*n* = 40) [BD]. Both Neo MTA Plus and Biodentine were prepared according to the manufacturer’s instructions and placed in the holes of the acrylic blocks. The samples were placed and covered with wet cotton pellets, then stored at 37 °C and 100% humidity for 72 h to complete setting/maturation. This schedule corresponds to a delayed restoration protocol (i.e., restoration performed well beyond the initial set) [29]. After maturation, bioceramic surfaces were prepared immediately before bonding by planing with 400-grit silicon-carbide (SiC) paper under water lubrication to obtain flat, parallel faces and to minimize off-axis loading during shear testing. The same preparation was applied to all groups. Specimens were gently rinsed and air-dried, and bonding was performed without additional storage. This step was used for experimental standardization of test faces and was not intended to replicate a clinical polishing procedure.

### 2.3. Randomization and Blinding

All acrylic blocks were randomly allocated to the experimental groups using a computer-generated random number sequence (https://www.random.org/). The restorative procedures and SBS tests were performed by a single operator who was aware of the study protocol but blinded to the group allocation. The failure mode analysis following SBS testing was conducted by an independent examiner who was blinded to the group identity to minimize observer bias.

### 2.4. Restorative Procedure

Following the preparation of the bioceramic materials, the NM and BD groups were randomly divided into four subgroups according to the restorative material used (*n* = 10 for each subgroup): nanofill composite (FU; Filtek Ultimate Universal Composite, 3M ESPE, St. Paul, MN, USA), giomer (BII; Beautifil II LS, Shofu Inc., Kyoto, Japan), bioactive restorative material (ACT; Activa BioActive-Restorative, Pulpdent, Watertown, MA, USA), and high-viscosity glass ionomer cement (GIC; Fuji IX GP Extra, GC, Tokyo, Japan). Each restorative material was applied by a single operator (as outlined in Table 1). For standardization purposes, acid etching and universal adhesive application were performed for all restorative materials, including those for which such steps are not recommended by manufacturers. A 37% phosphoric acid gel (Total Etch, Ivoclar, Vivadent AG, Schaan, Liechtenstein) was applied for 10–15 s and then rinsed for 5 s for all restorative material groups. The universal adhesive (G-Premio Bond, GC, Tokyo, Japan) was applied to the NM and BD groups for 10 s and light-cured using an LED light source with a power output of 1000 mW/cm^2^ (Valo Cordless, Ultradent, South Jordan, UT, USA) for 20 s. Phosphoric acid etching, adhesive application, and light-curing times were strictly followed as recommended by the manufacturers. The light-curing unit (Valo Cordless, Ultradent, South Jordan, UT, USA) was checked for output intensity using a radiometer before each application, and the intensity was consistently maintained at 1000 mW/cm^2^.

Restorative materials were then placed in the center of the NM and BD surfaces using a 2 mm diameter and 2 mm high cylindrical Teflon tube and light-cured for an additional 20 s. All restorative materials were applied as a single increment to achieve a standardized thickness of 2 mm, using a cylindrical Teflon mold. Application and light-curing were performed perpendicular to the specimen surface. All procedures were performed by a single experienced operator (with more than 5 years of laboratory research experience) to ensure standardization and reproducibility. The Teflon tubes were carefully removed, and all specimens were subjected to thermocycling to simulate oral aging conditions. Specimens were thermocycled (1000 cycles, 5–55 °C, 30-s dwell, ~10-s transfer) as a short-term aging protocol [30]. After thermocycling, the specimens were stored at 37 °C in 100% humidity for 24 h prior to SBS testing.

### 2.5. Shear Bond Strength (SBS) Testing

The samples were subjected to SBS testing using a universal testing machine (Instron, AGS-1000kGW; Shimadzu Corp., Tokyo, Japan). Each specimen was rigidly fixed in a custom-made alignment jig to ensure that the applied force was parallel to the adhesive interface. A knife-edge chisel-shaped plunger mounted on the movable crosshead of the machine was used to apply a load at a crosshead speed of 0.5 mm/min until failure occurred at the interface between the bioceramic and restorative material. Prior to testing, all samples were examined under a stereomicroscope (Leica MZ 125, Leica Microsystems, Wetzlar, Germany) at 20× magnification to exclude any specimens with visible cracks or defects. The maximum load at failure (in Newtons) was recorded, and SBS values were calculated in megapascals (MPa) by dividing the force by the bonding surface area (1 MPa = 1 N/mm^2^). All tests were performed at room temperature (22 ± 2 °C).

### 2.6. Stereomicroscopic Failure Analysis

Following the SBS testing, the debonded surfaces were qualitatively analyzed using a stereomicroscope (Leica Microsystems, Cambridge, UK) at 40× magnification to determine the mode of failure. The failure types were categorized as adhesive (failure at the interface between the bioceramic and restorative material), cohesive (failure within the bioceramic), or mixed (a combination of adhesive and cohesive failure). To enhance objectivity and reliability, all failure mode assessments were independently performed by two calibrated examiners who were blinded to group allocation. Any discrepancies in classification were resolved by consensus, and interexaminer agreement was evaluated using Cohen’s kappa statistic, confirming a high level of reproducibility.

### 2.7. Statistical Analysis

The normality of SBS data was assessed with the Shapiro–Wilk test and homogeneity of variances with Levene’s test. Between the two bioceramic groups (NeoMTA Plus vs. Biodentine), comparisons were performed with an independent-samples *t*-test. Within each bioceramic, restorative-material subgroups were compared by one-way ANOVA followed by Tukey’s HSD for pairwise contrasts when assumptions were met. In addition, distributions were visually inspected (Q–Q plots and box-plots) and a two-sided Grubbs test (α = 0.05) was used within each subgroup to screen for a single statistical outlier. Outliers, if any, were not removed a priori; to reduce the influence of dispersion we report robust summaries [median (IQR) and min–max] alongside mean ± SD, and we performed sensitivity analyses by re-estimating inferences after excluding the most extreme observation in each subgroup. Failure modes (adhesive, cohesive, mixed) were recorded as counts and percentages. Distributions between bioceramic groups (NeoMTA Plus vs. Biodentine) were compared using a chi-square test of independence (α = 0.05). The significance level was set at *p* < 0.05. All analyses were reviewed by a qualified biostatistician.

## 3. Results

The mean, minimum, maximum, and significance of the SBS values for the NM and BD groups are presented in Table 2. The mean SBS value of the BD group was significantly higher than that of the NM group (*p* < 0.001). Distribution diagnostics (Q–Q/box-plots) and a two-sided Grubbs test (α = 0.05) identified no statistical outliers in any subgroup (all *p* ≥ 0.05). A sensitivity analysis excluding the most extreme observation per subgroup did not alter the inference. Post-hoc sensitivity analysis showed that, with the achieved sample sizes, the minimum detectable effects at 80% power were f ≈ 0.39 for the within-bioceramic ANOVA and d ≈ 0.64 for the NeoMTA Plus versus Biodentine comparison.

The means and min/max SBS values for the subgroups within the BD groups are detailed in Table 3. SBS values of the restorative materials bonded to Biodentine revealed statistically significant differences among the tested groups (*p* < 0.05). FU exhibited the highest mean bond strength to BD, followed by BII; there was no statistically significant difference between their mean bond strengths (*p* > 0.05). By contrast, ACT and GIC exhibited significantly lower bond strengths to BD; there was no statistically significant difference between them (*p* > 0.05). Notably, a statistically significant difference was observed between the high-performing materials (FU and BII) and the low-performing materials (ACT and GIC) (*p* < 0.05).

The means and min/max SBS values for the subgroups within the NM groups are detailed in Table 4. SBS values of the restorative materials bonded to NeoMTA Plus did not reveal any statistically significant differences among the tested groups (*p* > 0.05). The lowest SBS was observed with BD–GIC and NM–GIC for both bioceramic bases.

### Failure Mode Analysis

**Figure 1:**
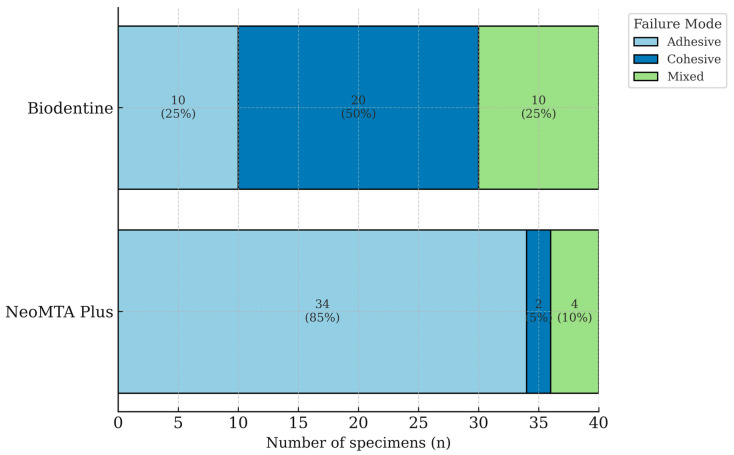
Distribution of failure modes (adhesive, cohesive, mixed) for NeoMTA Plus and Biodentine. Values inside the bars indicate counts (n) with corresponding percentages. Group distributions were statistically compared using a chi-square test of independence (2 × 3), showing a significant difference between NeoMTA Plus and Biodentine (*p* < 0.001).

The fracture patterns of the bioceramic–restorative interfaces are shown in Figure 1. Failure mode distribution differed markedly between the two bioceramic groups. In the NM group, adhesive failure was the predominant mode, observed in 85% of the specimens, indicating that most fractures occurred at the interface between the bioceramic and the restorative material. Only 5% of the NM specimens exhibited cohesive failure, and 10% displayed mixed failure patterns. By contrast, the BD group showed a significantly higher proportion of cohesive failures (50%). Adhesive and mixed failures were observed in 25% of the BD specimens each (Figure 1). The distribution of failure modes differed significantly between the two bioceramics (*p* < 0.001).

## 4. Discussion

The long-term success of vital pulp therapy relies not only on the biocompatibility and sealing ability of the bioceramic base material used but also on the quality of the bond it forms with the overlying restorative material [15]. Consistent with this objective, the present study evaluated the SBS of different restorative materials with two calcium silicate-based bioceramic materials. Based on the results, the null hypothesis—that both bioceramic materials would exhibit similar bond strength regardless of the restorative material used—was rejected.

Bond strength tests are one of the most frequently used in in vitro methods to predict the clinical success of dental materials. These tests are based on the principle that the stronger the adhesive bond between the restorative material and the biomaterial, the more resistant it will be to stresses caused by polymerization and functional forces in the oral environment. Shear and microtensile bond strength tests are among the most commonly applied methods for evaluating adhesive performance [31]. In this context, the SBS test used in this study was preferred as a practical and valid method to evaluate the durability of the interfacial bonds of bioceramics with restorative materials. However, bond strength is not an absolute material property; rather, it is a relative indicator influenced by numerous experimental variables such as surface preparation, type of adhesive agent, aging protocol, and timing of application [32]. Therefore, the comparative evaluations conducted under controlled conditions in our study are expected to provide insight into the potential clinical performance of the materials.

The results of this study demonstrated that Biodentine exhibited superior SBS to restorative materials compared with Neo MTA Plus. This finding is consistent with several previous studies evaluating the adhesive performance of bioceramics. Hursh et al. evaluated the bonding of four different bioceramic materials to dual-cure composite resin and reported that Biodentine provided significantly higher SBS values than Neo MTA Plus [27]. Similarly, Gürcan et al. reported that, especially when appropriate surface treatments were applied, Biodentine exhibited higher micro-shear bond strength than Neo MTA Plus and that surface preparation methods had a significant impact on this strength [33]. Xavier et al. also investigated the effect of four clinical variables on the bond strength of Neo MTA Pus and Biodentine, demonstrating that SBS values significantly increased in groups in which restoration was delayed and an additional hydrophobic bonding agent was used [2]. The superior bonding performance of Biodentine, may be attributed to its distinctive material characteristics, including a finer particle size, dense microstructure, and calcium chloride-accelerated setting reaction. These properties enhance calcium ion release and facilitate the formation of a stable and reactive interface, which is thought to promote effective interaction with methacrylate monomers [34,35]. However, a comparative study conducted by Falakaloğlu et al. compared the bonding performance of five bioceramics with a bulk-fill composite and found no statistically significant difference among the groups, except for TheraCal PT [36]. In a separate study, Suharwardy et al. compared the bond strength of self-adhering flowable and bulk-fill composites to four different pulp-capping materials, including Neo MTA Plus and Biodentine. Although the highest SBS value was obtained with the combination of Neo MTA Plus and SDR, the study emphasized that the type of restorative material had a direct impact on bonding performance, indicating that the bioceramic material alone was not the sole determining factor [37]. However, the Biodentine cohorts also showed markedly greater dispersion in SBS (SD = 11.35 MPa), which warrants comment on plausible sources of variability. The large spread for Biodentine is consistent with evidence that bond strength on calcium-silicate cements is highly maturation-dependent—values are lower and more variable at early time points and stabilize only after extended setting in aqueous media. In addition, microstructural heterogeneity (e.g., open/closed porosity and interfacial voids) documented for Bio-dentine can amplify specimen-to-specimen differences, while the material’s known handling sensitivity (powder–liquid ratio, mixing/activation, moisture control) can subtly alter setting kinetics and surface reactivity. Consistent with these mechanisms, our outlier screening and sensitivity checks confirmed that the comparative inferences remained stable despite this dispersion [38,39,40,41].

The bond strength between calcium silicate-based bioceramic materials and restorative materials can be significantly affected by differences in application protocols. Among these factors, adhesive strategy, timing of restoration, and surface treatment are key considerations. Krawczyk-Stuss et al. reported that prolonged acid application to the Biodentine surface and the use of self-etch adhesive systems significantly increased bond strength [42]. In the present study, to eliminate confounding variables and ensure methodological consistency, all restorative material groups underwent a standardized etching and bonding protocol. Another important factor influencing the adhesive performance of bioceramics is the maturation time of calcium silicate-based bioceramic materials. In an in vitro study conducted by Hoang-Thai Ha, the effect of Biodentine maturation time on its SBS to an overlying composite resin was evaluated. The study compared three different time intervals for applying the restorative material to the Biodentine surface: early (12 min), intermediate (72 h), and late (2 weeks). According to the results, SBS was significantly lower when the restoration was performed after 12 min whereas a notable increase in bond strength was observed in the 72-h and 2-week groups. Furthermore, the SBS value in the 2-week group was also reported to be significantly higher than that of the 72-h group [29]. These findings indicate that placing a restoration on bioceramic cements before they have adequately matured may negatively affect bonding success. These findings support our protocol of allowing adequate maturation time before restorative placement to optimize adhesive outcomes. However, variations in surface treatment protocols and restoration timing across studies make direct comparison of the results challenging.

The bonding performance of restorative materials in combination with Biodentine demonstrated significant differences in SBS values. The highest SBS was observed in the nanofilled composite, followed by the giomer; however, no statistically significant difference was found between these two groups. Similarly, Durmazpinar et al. evaluated the micro shear bond strength (μSBS) of MTA to four restorative materials—nanohybrid resin composite, giomer, alkasite, and ormocer. A statistically significant superiority of alkasite over all other groups was observed whereas nanohybrid composite, giomer, and ormocer showed comparable performance with no significant differences [43]. The comparable performance of giomer and composite may be explained by their resin-based matrices, which enable similar micromechanical and adhesive interaction profiles with calcium silicate bioceramics, particularly under a standardized total-etching and universal adhesive protocol. However, Ajami et al. compared the SBS of composite resin and giomer to MTA at different time intervals. They found that composite resin had significantly higher bond strength than giomer at both 2.45 h and 3 days after mixing MTA [44]. This contrasts with the findings of this study, possibly due to differences in the bioceramic substrate or bonding conditions.

In the present study, although Biodentine exhibited significant variations in SBS depending on the type of restorative material, this pattern was not observed with Neo MTA Plus. This finding suggests that, unlike Biodentine, the bonding performance of Neo MTA Plus appears to be less dependent on the choice of restorative material. Biodentine has a short early setting time (≈12 min), which facilitates earlier maturation of the surface for bonding [45]. By contrast, MTA-type cements (the class that includes NeoMTA Plus) hydrate more slowly, a difference that has been repeatedly noted in comparative reviews of Biodentine versus MTA [46]. NeoMTA Plus also differs in formulation—It is supplied as a powder–gel system with tantalum oxide radiopacifier—which can yield a surface chemistry distinct from Biodentine [47]. The absence of significant SBS differences over NeoMTA Plus likely reflects a comparatively less Ca-rich, more passivated surface during early maturation (~72 h). Lower Ca^2+^ availability—partly due to hydration/carbonation at the outer layer and the use of non-calcium radiopacifier (tantalum oxide)—can limit formation of stable MDP–Ca salts and other substrate-specific chemical bonds. With fewer reactive sites, the interface becomes adhesive-dominated rather than substrate-sensitive, so different restorative chemistries converge to similar bond strengths. The standardized surface preparation and constant adhesive protocol of the present study may have further minimized variability, reinforcing this pattern [28,47].

In the Biodentine group, both Activa BioActive-Restorative and high-viscosity glass ionomer cement exhibited significantly lower SBS values than the nanofilled composite and giomer subgroups. Despite applying phosphoric acid etching and a universal adhesive as recommended by the manufacturer, Activa BioActive-Restorative did not achieve the expected bonding performance. Martínez-Sabio et al. compared the SBS and microleakage of Activa BioActive-Restorative, two bulk-fill composites, and a conventional composite at multiple time intervals, reporting no statistically significant differences in SBS among the materials [48]. Notably, no statistically significant difference was observed between Activa and high-viscosity GIC in our study, indicating that Activa BioActive-Restorative performed at a bonding level similar to conventional GIC. By contrast, Ergül et al. investigated the SBS between various calcium silicate-based materials and three types of glass ionomer-based restorative materials. They reported that Activa BioActive-Restorative achieved higher bond strength than conventional GIC, which differs from the results of the present study [49]. Although Activa BioActive-Restorative is marketed as a bioactive material, its performance in this study was comparable to that of the conventional high-viscosity GIC. Activa’s bioactivity is sensitive to environmental conditions, such as moisture control and pH fluctuations; under standardized in vitro conditions, its ion-exchange potential may be less pronounced. In addition, the relatively high resin-matrix content and filler loading can limit direct ionic interaction with calcium-silicate substrates compared with conventional glass ionomers. Finally, short-term shear bond testing primarily captures immediate adhesion rather than longer-term mineralizing behavior; thus, the similarity to GIC observed here may reflect the constraints of a short-term protocol rather than the absence of bioactivity [49,50,51]. Likewise, Kenchappa et al. found that composite resin achieved the highest SBS to Biodentine, followed by resin-modified GIC whereas Fuji IX GIC showed the lowest values, suggesting that Biodentine bonds more effectively to resin-based materials than to conventional glass ionomer formulations and that Biodentine adheres more effectively to resin-based materials compared with conventional glass ionomer formulations [52]. These discrepancies may be attributed to methodological variations such as differences in the type of substrate, surface conditioning protocols, adhesive systems, and aging procedures.

Failure mode analysis provides qualitative insights into the nature of the bond between restorative materials and bioceramic substrates. In the present study, the distribution of failure modes largely corresponded to the measured SBS values, providing additional insight into the quality of the adhesive interfaces. The Biodentine group predominantly exhibited cohesive failures, followed by equal proportions of adhesive and mixed failures. In general, higher bond strength values were associated with a predominance of cohesive failures within the restorative material or mixed failures, which indicates a stronger adhesive interface capable of withstanding stress before the material itself fractures [44]. By contrast, the NeoMTA group demonstrated a markedly higher incidence of adhesive failures, with mixed and cohesive failures occurring at substantially lower rates. The predominance of adhesive failures has been linked to weaker bonding performance and insufficient micromechanical or chemical retention between the materials [31]. These findings are partially consistent with the study by İpek et al. in which cohesive and mixed failures predominated in both the Biodentine and MTA Cem LC groups whereas the NeoPutty group showed a predominance of adhesive failures [31]. Similarly Hursh et al. reported that 90% of the specimens exhibited either cohesive or mixed fracture patterns in the Biodentine group [27]. Gürcan et al. also observed a predominance of cohesive failures in Biodentine, which is consistent with our results. However, in contrast to this study, they reported an equal distribution of adhesive, cohesive, and mixed failures for the NeoMTA Plus group [33]. This difference may be related to variations in experimental design, surface treatment protocols, or subtle differences in material handling that could influence the bonding interface.

This in-vitro study used a unified bonding protocol to maximize internal validity; therefore, results reflect performance over an identically primed bioceramic surface and may not directly generalize to adhesive-free placement of GIC or Activa or to manufacturer-specific conditioners. The model did not reproduce full intraoral complexity (moisture, saliva/biofilm, cyclic loading), and aging was limited to 1000 thermocycles, so long-term durability remains uncertain. Surface preparation with 400-grit SiC may have influenced microtopography; however, the same preparation across groups preserves between-group comparisons. Interfacial mechanisms were not examined with high-resolution microscopy/spectroscopy, and only one brand per material class was tested; batch numbers were not recorded, limiting generalizability and traceability. Future studies should include material-specific protocols, extended aging/fatigue, and interface-level characterization.

## 5. Conclusions

Within the limitations of the present study, Biodentine exhibited significantly higher SBS with restorative materials than NeoMTA Plus and showed a failure-mode profile dominated by cohesive fractures, consistent with a stronger and more resilient adhesive interface. Over Biodentine, the nanofilled composite and the giomer achieved the highest and statistically comparable bond strengths whereas the Activa BioActive-Restorative and the high-viscosity glass ionomer performed at substantially lower levels. By contrast, restorations placed over NeoMTA Plus showed uniformly lower bond strengths with no significant differences among materials, and failures were predominantly adhesive, indicating a weaker interfacial interaction. Taken together, these results suggest that when a calcium-silicate base is required, Biodentine combined with a resin-based restorative (nanofilled composite or giomer) offers superior bonding stability compared with NeoMTA Plus. Given their comparable bond strength to Biodentine, giomers, being biointeractive materials, may serve as a clinically viable alternative to resin composites in scenarios in which enhanced ion release and bio-interactivity are desired. Future research should verify these findings under clinical conditions and systematically test protocol variables (maturation time, surface conditioning, and adhesive strategy) to optimize the durability of bioceramic–restorative interfaces.

## Figures and Tables

**Table 1:** The bioceramics and restorative materials used in the study.

Material	Manufacturer	Composition	Application	Lot No./ Expiry
NeoMTA Plus	Avalon Biomed Inc., Houston, TX, USA	Powder: Tricalcium silicate, dicalcium silicate, tantalite, calcium sulfate and silica; Liquid: salt-free polymer gel	Mix powder and liquid (1:3). Place it into the cavity. Set for 50–60 min.	NA
Biodentine	Septodont, Saint-Maur-des-Fossés, France	Powder: Tricalcium silicate, dicalcium silicate, calcium carbonate, zirconium oxide, calcium oxide, iron oxide; Liquid: calcium chloride, a hydrosoluble polymer and water	Capsule mixed in amalgamator 30 s, place, sets in 6.5–12 min	NA
Filtek Ultimate Universal Composite	3M ESPE, St. Paul, MN, USA	Matrix: Bis-GMA, UDMA, TEGDMA, bis-EMA, PEGDMA; Fillers: 63.3% wt silica/zirconia (nanofiller), glass ceramic	Etch (10–15 s), rinse/dry, bond (G-Premio), light-cure 10 s, apply composite, light-cure 20 s at 1000 mW/cm^2^ standard power.	NA
Beautifil II (A2)	Shofu Inc., Kyoto, Japan	Matrix: Bis-GMA, UDMA, TEGDMA, Bis-MPEPP; Filler: 83.3% wt S-PRG (surface pre-reacted glass)	Etch (10–15 s), rinse/dry, bond (G-Premio), light-cure 10 s, apply composite, light-cure 20 s at 1000 mW/cm^2^ standard power.	NA
Activa BioActive Restorative (A2)	Pulpdent Corporation, Watertown, MA, USA	Diurethane and other methacrylates, modified polyacrylic acid, 55% bioactive glass, sodium fluoride	Etch (10–15 s), rinse/dry, bond (G-Premio), light-cure 10 s, apply composite, light-cure 20 s at 1000 mW/cm^2^ standard power.	NA
Fuji IX GP Extra	GC Corporation, Tokyo, Japan	Powder: Fluoroaluminosilicate glass, polyacrylic acid, tartaric acid; Liquid: polybasic acid, water	Used in capsule form. Etch for 10–15 s, rinse for 5 s, and dry. Applied to bioceramic surface.	NA
Total Etch	Ivoclar Vivadent AG, Schaan, Liechtenstein	37% phosphoric acid gel	Apply 10–15 s, rinse 5 s and dry	NA
G-Premio Bond	GC Corporation, Tokyo, Japan	4-MET, 10-MDP, MDTP, phosphoric acid monomer, acetone, water, photoinitiators, colloidal silica	Apply for 10 s, air dry 5 s, light-cure 10 s at 1000 mW/cm^2^ standard power.	NA

UDMA, urethane dimethacrylate; Bis-GMA, bisphenol-A glycidyl methacrylate; Bis-EMA, ethoxylated bisphenol-A dimethacrylate; Bis-MPEPP (manufacturer’s proprietary monomer); TEGDMA, triethylene glycol dimethacrylate; 4-MET, 4-[2-(methacryloyloxy)ethoxycarbonyl] phthalic acid; 10-MDP, 10-methacryloyloxydecyl dihydrogen phosphate; MDTP, methacryloyloxydecyl thiophosphate; S-PRG, surface pre-reacted glass-ionomer filler; NA, not available (lot numbers were not recorded during experimentation; all products were within expiry and sourced from official distributors).

**Table 2:** Shear bond strength values (MPa) of bioceramics.

Groups	Mean ± SD (MPa)	Min/Max (MPa)	*p*
NM	5.24 ± 3.08 ^a^	1.24/11.58	0.001
BD	11.52 ± 11.35 ^b^	2.20/41.58	

NM, NeoMTA Plus; BD, Biodentine. Different superscript letters in the same column mean statistically significant differences (*p* < 0.05). No statistical outliers were detected by the Grubbs test (α = 0.05); *p* values from independent-samples *t*-test (NM vs. BD).

**Table 3:** Shear bond strength values (MPa) of restorative materials to Biodentine.

Subgroups	Mean ± SD (MPa)	Min/Max (MPa)
FU	20.04 ± 9.98 ^b^	10.34/37.31
BII	19.39 ± 12.55 ^b^	7.16/41.58
ACT	3.89 ± 1.20 ^a^	2.25/6.05
GIC	2.76 ± 0.41 ^a^	2.20/3.40

FU, Filtek Ultimate Universal composite; BII, Beautifil II; ACT, Activa BioActive-Restorative; GIC, Fuji IX GP Extra high-viscosity glass ionomer cement. Different superscript letters indicate statistically significant differences among subgroups within the same column according to Tukey’s HSD test (*p* < 0.05).

**Table 4:** Shear bond strength values (MPa) of restorative materials to NeoMTA.

Subgroups	Mean ± SD (MPa)	Min/Max (MPa)
FU	5.67 ± 4.06 ^a^	1.24/11.58
BII	7.05 ± 2.50 ^a^	3.08/10.05
ACT	6.07 ± 1.80 ^a^	3.20/8.58
GIC	2.18 ± 0.54 ^a^	1.40/3.00

FU, Filtek Ultimate Universal composite; BII, Beautifil II; ACT, Activa BioActive-Restorative; GIC, Fuji IX GP Extra high-viscosity glass ionomer cement. Identical superscript letters indicate no statistically significant differences among subgroups within the same column according to Tukey’s HSD test (*p* < 0.05).

## Data Availability

The data used to support the findings of this study can be made available upon request to the corresponding author.
